# Regenerative medicine: characterization of human bone matrix gelatin (BMG) and folded platelet-rich fibrin (F-PRF) membranes alone and in combination (sticky bone)

**DOI:** 10.1007/s10561-021-09925-9

**Published:** 2021-06-01

**Authors:** Lajos Csönge, Ágnes Bozsik, Zoltán Tóth-Bagi, Róbert Gyuris, János Kónya

**Affiliations:** 1 Petz Aladár University Teaching Hospital, West Hungarian Regional Tissue Bank, 2 Vasvári, Győr, 9024 Hungary; 2 Private Praxis, Budapest, Hungary; 3 Private Praxis, Eger, Hungary; 4 Dent-Art-Technik Ltd, Győr, Hungary

**Keywords:** Regenerative medicine, F-PRF membrane, BMG, Sticky bone, Implant dentistry

## Abstract

During the last two decades autologous platelet and leukocyte rich products (PRP; PRF), opened new perspectives in regenerative medicine. In particular regenerative dentistry played a pioneer role in the application of these products in bone regenerative cases. Many aspects of cytokines, such as, growth factor release, blood cell content and its characterization were reported, but some practical questions are still unanswered in the preparation of PRF membranes and sticky bones. A new folding technique was introduced that created a good quality, pliable, and strong F-PRF membrane with a dense fibrin network and more homogenous blood cell distribution. F-PRF produced a very promising sticky bone combined with human freeze-dried cortical bone matrix gelatin (BMG). There hasn’t been much focus on the quality and character of the applied bone and the optimal membrane/bone particle ratio has not been reported. A 0.125 g BMG/ml plasma (1 g/8 ml) seems like the ideal combination with maximal BMG adhesion capacity of the membrane. Particle distribution of BMG showed that 3/4 of the particles ranged between 300–1000 µ, the remnant 1/4 was smaller than 300 µ. The whole F-PRF membrane and its parts were compared with conventional A-PRF membrane concerning their resistance against proteolytic digestion. The F-PRF was superior to A-PRF, which dissolved within 4–5 days, while F-PRF was destroyed only after 11 days, so this provides a better chance for local bone morphogenesis. The F-PRF pieces had similar resistance to the whole intact one, so they can be ideal for surgical procedures without risk of fast disintegration.

## Introduction

Recently, a rapidly emerging number of articles have been published on autologous platelet rich blood products in tissue and bone regeneration, especially in regenerative dentistry (Chaparro et al. 2016; EDQM 2019; Miron et al. 2017; Theys et al. 2018; Dawood et al. 2018; Crisci et al. 2019; Kemmochi et al. 2018; Grecu et al. 2019; Isobe et al. 2017).

The most challenging issues in oral implantology are alveolar ridge deficiencies and bone loss, whether it is the result of physiologic bone resorption, trauma, tooth extraction, or periodontal disease. In the absence of good quality and optimal quantity bone tissue, a stable implant cannot be achieved in the long term. In extreme cases of bone resorption, implantation is not possible (Borie et al. 2015).

Several techniques for platelet concentrates have been introduced in the surgical field for the acceleration of tissue regeneration. Two main groups of platelet rich products can be distinguished: 1) first generation platelet rich plasma (PRP) products with anticoagulants (Marx et al. 1998) and 2) second generation platelet rich fibrin (PRF) products without anticoagulants (Choukroun et al. 2006).

Depending on the leukocyte fibrin content, platelet concentrates can be classified into different categories: leukocyte and platelet-rich plasma (L-PRP); pure platelet rich fibrin (P-PRF); leukocyte and platelet-rich fibrin (L-PRF); injectable i-PRF; advanced A-PRF; autologous albumin gel and platelet rich fibrin (Alb-PRF); etc.

Many articles describe these different PRF membranes. Their properties which have been reported, include platelet number, cell number and their distribution in membranes, biomechanical properties, and cytokin distribution and their release over time (Takahashi et al. 2019; Ehrenfest et al. 2018; Kobayashi et al. 2020; Isobe et al. 2017; Kardos et al. 2018). This report is not going to recapitulate these findings.

The Platelet and Leukocyte Rich Fibrin membranes (L-PRF) containing concentrated platelets release many cytokines and growth factors (e.g., PDGF, EGF, vascular endothelial growth factor-VEGF, TGF), which play a key role in tissue and bone regeneration. 10^6^ /µl platelets are likely and are in the therapeutically effective range, it is 4–5 times higher than baseline platelet count values in whole blood (Everts et al. 2006).

These membranes are prepared in centrifuge tubes alone or with metal kits. In spite of the large amount of research on this, a routine significant amount of important information is still missing that concerns the ideal preparation of the product in daily practice.

The different particulated bone grafts with PRF creates a so called “sticky bone” or “gummy bone”, which is a pliable, flexible, biologically active bioscaffold (Sohn et al. 2015).

Combining human freeze-dried particulated Bone Matrix Gelatine (BMG) allograft with autologous folded F-PRF is a new combination in this field.

BMG produced by the West Hungarian Regional Tissue Bank in Győr (WHRTB) has been using this for 27 years with excellent clinical outcomes both in orthopaedic or oral surgery cases (Eitler et al. 1997).

The quality and character of the applied bone has not been focused on in the processing of sticky bone. Cortical bone was chosen as raw material because it produced better clinical results than cancellous bone, and cortical bone contains more bone morphogenetic proteins (BMPs) than cancellous bone per weight unit. BMPs play a crucial role in the initial steps of bone tissue morphogenesis (Urist et al. 1983); however, the ideal size of bone particles used to fill bone defects is ill-defined and a disputed issue (Malinin et al. 2007; Temple and Malinin 2008). A clinical trial is still running to find the ideal bone allograft particle size in grafted extraction sockets and edentulous ridges (Abou-Arraj 2020–2021).

In addition, the ideal membrane/bone ratio is still unknown and nobody has addressed the question.

The goal of the report is the characterization of some special properties of the new folded membrane and BMG alone or in combination.

## Materials and methods

### F-PRF membrane and sticky bone preparation. Membrane resistance against proteolytic digestion. Distribution of BMG particles

*Blood collection* The blood was collected from 6 volunteer donors. Blood samples were collected with the informed consent of six donors. Experiments were in accordance with the ethical standards of the Regional Research and Ethic Commitee.

Whole blood was drawn by venipuncture from cubital veins into 55 (15 + 40) nine ml vacutainer tubes without any chemicals (Vacuette, Gerner BioOne). Proper hydration is a very important issue to facilitate drawing blood so drinking at least 500–600 ml water was recommended within an hour prior to blood donation. For optimal membrane and tissue repair we need to get as much cytokine as possible from peripheral blood so not only the veins but also the brachial artery was strongly pressed with a tourniquet for 30 s. During transient short hypoxia of the arm a lot of cytokines are released into the vascular system to repair the “injured” area, as a result it was not a simple analytical taking of blood.

*Cell separation* ue to the short lifespan of some cytokines, growth factors and clotting of the blood sample, cell separation has to be started as soon as possible, within 2 min maximum. Cell separation was performed by Steinberg centrifuge (CGOLDENWALL 80–2) at 375 revolutions per minute (RPM) for 10 min to prepare F-PRF.

Determination of bone particle adhesion capacity of F-PRF of 5 donors (3 male and 2 female healthy donors, between 45–59 years, age average: 50.8 years). After cell separation, the supernatant quantity was measured in milliliters. 4 ml of plasma could be removed from each tube and 8 ml plasma of 2 tubes was put into 4 rectangular metal jars manufactured for F-PRF (Dent-Art-Technik Győr, Hungary). After 8 min at room temperature, fibrin filaments started to appear. Different quantites of BMG were added to each gelatinous material: 0.5,1,2,3 g bone/8 ml of plasma clot to determine the particle adhesion capacity of the membrane. In the early gelatinous stage the membrane was folded 4–5 times with forceps by seizing the corners of the membrane to enmesh and entrap the bone particles and blood cells completely within the dense fibrin network and minimize particle loss. The membrane could be formed into different shapes by squeezing out the fluids present in the fibrin clot using a stainless steel compression device.

The bone particle distribution was determined by macroscopic assessment and the adhesion capacity of the membrane was measured by weighing the surplus of BMG particles that were not absorbed by the membrane. The wet weight of BMG is 32.5% higher than the freeze dried one according to our assessments. This fact was considered at the weighing and calculation of surplus bone particles.

*Resistance of F-PRF and A-PRF membranes against enzymatic digestion *The objective of this experiment was to mimic the clinical conditions and assess the resistance of membranes without bone particles against enzymatic protein digestion after transplantation. 60 ml of plasma (15 tubes, 4 ml in each tube) was used to compare resistance of 2 different PRF membrane types of one donor:

1. The F- PRF membrane was prepared and kept a) in one big piece (20 ml plasma) and b) a similar membrane (20 ml plasma) was prepared by cutting it into 5 uniform pieces.
2. For A-PRF (advanced PRF) preparation the PRF Duo centrifuge (Nice, France) at 1300 RPM for 8 min was applied using 20 ml of plasma in 5 tubes of blood. The membrane was prepared by a commercially available A-PRF preparation metal kit. The membrane was divided into 5 uniform pieces.

All the fresh membranes were weighed then kept in Dulbecco’s Minimal Essential Medium (DMEM) containing 0.1% (v/v) trypsin (Sigma, USA) and 0.4 mg/ml EDTA at cell culture conditions in HeraCell 150 CO_2_ thermostate (Heraeus, Germany) at + 37 °C in 5% CO_2_ to feed the membranes. DMEM with trypsin was replaced every day. The weights of membrane pieces were assessed every 24 h using a laboratory scale (Kern, Germany) for 11 days.

The big membrane (1a) was stored in an 8 ml medium-enzyme mixture in a plastic Petri dish and all the small pieces (1b and 2) were in a 1 ml mixture in a 24 cell well plate.

F-PRF was also examined using conventional histology and immunohistochemistry for VEGF identification.

*Determination of BMG particle sizes and their distribution* BMG was manufactured according to the modified Urist’s method (Eitler et al. 1997). The BMG was processed from the femur diaphysis of a 25 year old male multiorgan and tissue donor at WHRTB. Cortical bone pieces were ground using a MF10 basic micro fine grinder (IKA, Germany) to create cortical bone powder for the raw material. An interchangeable sieve with 1 mm holes ensured maximum particle size filtering. After complex processing the product was sterilized and freeze dried. A 1 cm3 of BMG has ~ 1 g dry weight.

The freeze dried bone matrix gelatin powder particles were separated with a high performance vibratory sieve shaker applying 1 mm amplitude for 10 min (Retsch AS 200, Germany). It offers fast determination of quantitative particle size distribution. The measurement range was 0–1000 µ using 50–100–200–300–500–800-1000 pore size sieves.

## Results

The folded membranes were strong, flexible, and pliable. The findings were similar in each case when BMG was added:

0.5 g/8 ml (0.0625 g/ml): Particles were distributed homogenously in the membrane. There were large gaps among particles.

1 g/8 ml (0.125 g/ml): the membrane was saturated pretty homogenously, there were no big gaps among particles and there were no particle clusters.

2 g/8 ml (0.25 g/ml): the membrane was densely saturated inhomogeneously and bone particle clusters formed. There was 0.954 g (SD: 0.106) calculated average dry weight surplus which was not absorbed by the membrane.

3 g/8 ml (0.375 g/ml): the membrane was densely saturated, there were large clusters like in the 0.25 g/ml case with 2.1 g (SD:0.28) average excess bone particles.

**Fig. 1:**
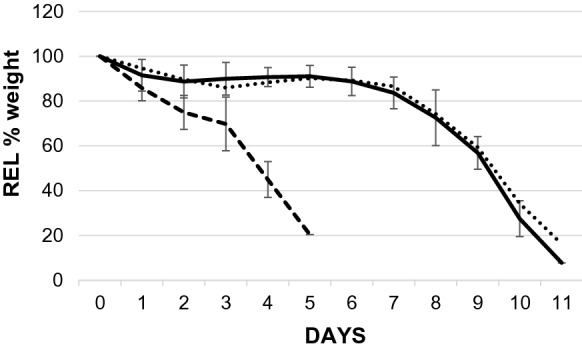
Comparison of enzymatic protein digestion resistance of the 3 membranes. Weight of samples were expressed in relative %. Initial weight was set as 100%. There was a significant difference between F-PRF and A-PRF (*p* < 0.01) from day 1. A-PRF: rapid enzymatic destruction could be seen. There was a remarkable resistance against enzymatic digestion for 1 week in F-PRF. During 2nd week the dissolving was accelerated. Dotted line: 1a folded PRF (F-PRF) forms intact big membrane (~ 676 mg *n* = 1). Thin line: 1b F-PRF membrane cut into 5 pieces (*n* = 5). Spaced line: 2 A-PRF membrane cut into 5 pieces (*n* = 5)

The F-PRF and A-PRF showed different decomposition properties (see Fig. 1). No difference could be seen between intact and cut F-PRF, but there was a significant difference between the F-PRF and A-PRF membrane. The F-PRF was obviously superior to the A-PRF. After 5 days only one of the A-PRF of the 5 remained.

**Fig. 2:**
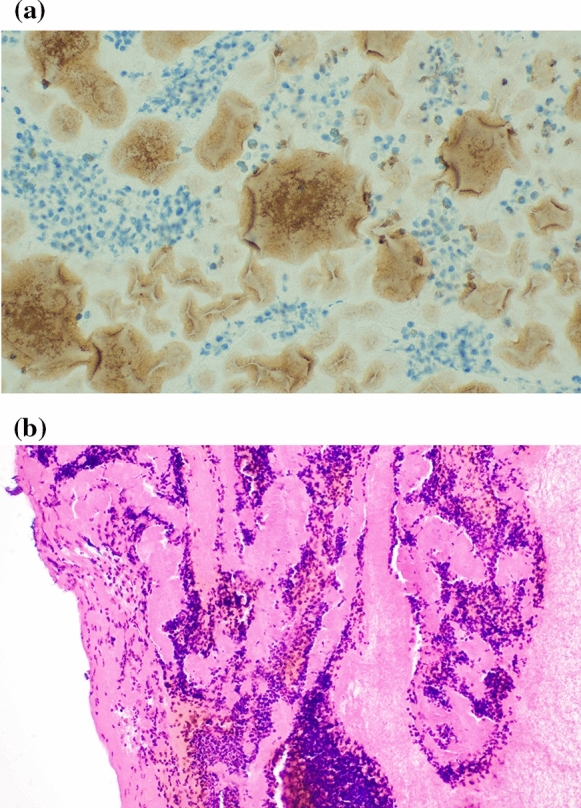
**a** Immunhistochemistry: strong VEGF positivity can be seen around platelet islands (brown). The platelets are surrounded by leukocyte groups (blue). 400 × magnification. **b** Leukocyte groups are separated by fibrin bunches after folding process. Hematoxylin–eosin staining. 200 × magnification

Leukocyte and platelet groups were situated homogenously in groups in folded F-PRF (see Fig. 2a and b). Strong VEGF positivity was also found.

The BMG consisted of different particle fractions (see Table 1). 74% of the powder was composed of particles ranging 300–1000 µ. The remnant 26% contained particles ranging 0–300 µ.

**Table 1:** Composition of BMG (particle fractions)

μ	Weight (gr)	%
> 1000	0	0.0
800–1000	1.95	0.8
500–800	122.82	51.5
300–500	51.73	21.7
200–300	17.17	7.2
100–200	22.06	9.2
50–100	13.77	5.8
< 50	9 3	3.8
Sum	238.5	100

## Discussion

During the last decade the clinical application of platelet rich blood products like PRFs and sticky bone became very popular, especially in dental implantology. Good quality bone is crucial for stable implants.

The combination of blood derivative membranes and allograft freeze dried bone products became a reasonable clinical process in many disciplines.

In spite of many articles in this field our knowledge is still not consistent enough. There are some uncertainties due to graft properties, e.g., platelet number, etc., so the processes can be standardized but the products can’t. Some problems have not been solved that have arisen during daily practice, e.g., bone/membrane ratio and the bone particle absorption capacity of platelet rich membranes. Everts et al. reported that human bone particles, and osteoblasts are ideal for creating a very strong structure with thrombin and platelets, and maybe this is an explanation for the good adhesion. PRF fragments serve as a biological connector between bone particles (Everts et al. 2006).

On the one hand, the saturation of membranes by BMG particles seems important for ideal osteogenesis but on the other hand, the effect of densely packed particle clusters is not clear. Further experiments have to be performed to judge their favorable, unfavorable, or neutral effect on bone morphogenesis.

A 0.125 g/ml BMG-plasma ratio is recommended according to our findings. This composition is dense and homogenous enough and it provides the maximum bone adhesion capacity in F-PRF membrane without particle clusters.

The folding method has produced a much more homogenous cell distribution than conventional PRF that has been processed in a centrifuge tube; where blood cells have been separated in different layers and zones (Crisci et al. 2019).

BMG was chosen instead of cancellous chips or classic DBM (demineralized bone matrix) because the authors have very good long-term experiences using human freeze-dried BMG with its concentrated protein content in bone loss cases. Cortical bone contains much more bone morphogenetic proteins than cancellous bone. Spence’s earlier report confirmed that cortical bone is superior to cancellous one (Spence et al. 1976), and freeze dried allograft induces faster healing of osseous defects than a frozen one (Malinin et al. 2007).

Mechanical and osteoinductive properties of BMG with F-PRF provided us with reasonable clinical expectations. These expectations were realized in clinical practice and will soon be published.

The ideal bone particle size is still a controversial issue. 3/4 of BMG consists of 300–1000 µ particles, 1/4 contains particles between a 0 to 300 µ range according to our findings. Malinin et al. reported that cortical bone allografts with particle sizes in the range of 300–90 µm were comparable to autograft and showed the best results in baboons (Malinin et al. 2007). In metadiaphyseal surgical bone defects incorporation and consolidation were observed in over 90% of patients applying 250µ cortical bone allografts particles (Temple and Malinin 2008). Some authors found better bone augmentation results using larger particles (1–2 mm) in comparison to smaller ones (0.25–1 mm) in animal models (Yamada et al. 2018). The determination of ideal particle size in extraction socket cases is still running in a clinical trial (Abou-Arraj 2020–2021).

Better enzymatic resistance of folded F-PRF was found in the present experiment compared to the traditional A-PRF preparation method. It seems the folding procedure increases the resistance and density of fibrin network serving like biological autoscaffold and showed better resistance against enzymatic digestion of membrane proteins. It provides more time for longer cytokine and growth factor release from entrapped blood cells and a chance for good osteogenesis.

In spite of the expectation there was no difference between the whole intact F-PRF membrane and its pieces. The possible advantage is that the membrane can be cut into ideal pieces for surgical procedures without the risk of fast disintegration.

The F-PRF process obviously requires manual manipulation, so it needs to be performed under aseptic conditions clinically. After the early introduction of open-system experts were alerted to safety issues (O’Connell 2007). During the last decade the huge and emerging number of clinical cases did not confirm the worries. There were no available references on the serious adverse event (SAE) or serious adverse reaction (SAR) in the application of platelet and leukocyte rich products.

Many question still remain to be answered and further in vitro and clinical investigations are required to find optimal methods in sticky bone processing and its clinical application.

## Data Availability

The datasets generated during and analysed during the current study are available from the corresponding author on reasonable request.
